# “His mind will work better with both of us”: a qualitative study on fathers’ roles and coparenting of young children in rural Pakistan

**DOI:** 10.1186/s12889-018-6143-9

**Published:** 2018-11-20

**Authors:** Joshua Jeong, Saima Siyal, Günther Fink, Dana Charles McCoy, Aisha K. Yousafzai

**Affiliations:** 1 Department of Global Health and Population, Harvard T.H. Chan School of Public Health, 665 Huntington Avenue, 11th floor, Boston, MA USA; 2 Aga Khan University, Karachi, Pakistan; 3 Swiss Tropical and Public Health Institute, Basel, Switzerland; 4 University of Basel, Basel, Switzerland; 5 Harvard Graduate School of Education, Cambridge, MA USA

**Keywords:** Pakistan, Fathers, Parenting, Early child development, Qualitative research

## Abstract

**Background:**

Parents are the primary providers of nurturing care for young children’s healthy early development. However, the literature on parenting in early childhood, especially in low- and middle-income countries, has primarily focused on mothers. In this study, we investigate how parents make meaning of fathers’ parenting roles with regards to their young children’s early health and development in rural Pakistan.

**Methods:**

Data were collected between January and March 2017 through in-depth interviews with fathers (*N* = 33) and their partners (*N* = 32); as well as separate focus group discussions with fathers (*N* = 7) and mothers (*N* = 7). Data were analyzed using thematic content analysis.

**Results:**

Parents described a distinct division of roles between fathers and mothers; and also several shared caregiving roles of fathers and mothers. Specifically, parents highlighted aspects of fathers’ coparenting and several common ways by which fathers supported their partners. We found that these gendered divisions in parenting roles were strongly embedded within a complex network of interacting factors across the individual, family, and sociocultural contexts of the study community.

**Conclusions:**

Our findings suggest a more family-centered conceptualization of fatherhood during early childhood that encompasses both fathers’ direct engagement with their young children and their indirect contributions through coparenting, while recognizing a variety of contextual systems that shape paternal parenting. Future parenting interventions that reflect the lived experiences of both fathers and mothers as parents and partners may further enhance the nurturing care environments that are critical for promoting healthy early child development.

## Background

Positive parenting by fathers and mothers profoundly promotes healthy development of children and families [1, 2]. Parents are especially critical during the earliest years of childhood, when children are most dependent on adults to meet their basic needs and provide warmth, affection, protection, and care that are essential for healthy early childhood development (ECD) [3]. Even though the majority of the world’s children grow up in families with both their biological parents [4] most of the parenting literature has focused on mothers and the mother-child dyadic relationship. Relatively little work has considered fathers’ experiences within families or their caregiving roles for promoting children’s early health and development [5, 6].

A recently growing body of evidence has emphasized how paternal behaviors and characteristics independently predict a wide range of ECD outcomes. For example, studies have found that fathers’ sensitivity and cognitive stimulation [7], physical play [8], and education [9] influence ECD outcomes, controlling for the same maternal characteristics. Moreover, fathers are important for coparenting, or the support that each parent provides for one another, such as through sharing responsibilities or emotional care [10]. Emerging studies have documented how fathers’ coparenting relationships shape maternal parenting behaviors as well as children’s early development outcomes [11, 12].

While this body of evidence has raised the visibility and expanded our perspective on paternal parenting, the majority of the literature on paternal roles in early childhood is based in the United States and other high-income countries (HICs). Much remains unknown about fathers’ roles for promoting ECD in low- and middle-income countries (LMICs), including Pakistan. Particularly considering recent demographic, socioeconomic, and cultural shifts that have enabled greater opportunities for women and altered household divisions of labor across LMICs [13], an investigation of fathers’ contemporary roles in promoting children’s ECD is critically needed across diverse cross-cultural populations around the world.

### Fatherhood in global contexts and specifically in Pakistan

Nurturing caregiving practices have been generally described to be much lower in LMICs than HICs for a variety of reasons. First, abject conditions of poverty disproportionally undermine parents’ caregiving in LMICs [14]. Second, young children in LMICs are more likely exposed to a host of additional risk factors that immediately threaten mortality and malnutrition, thereby contributing to parental care that focuses more on child survival than child development and early learning [15]. Moreover, constraints to fathers’ and mothers’ care practices in LMICs are more likely compounded by inadequate and poorer health, education, and social protection services; and lack of early child and family policies in LMICs [16].

In addition to these broad contextual differences between LMIC and HICs, there are also substantial variations across LMICs that may uniquely differentiate fathers’ and mothers’ parenting within and between LMICs around the world. Specifically in Pakistan, households tend to be large (7 persons on average) and commonly of joint family structures [17]. Consequently, fathers’ roles in Pakistan may be importantly shaped by other family members (i.e., aunts, uncles, grandparents) who live together and share key childrearing roles [18]. Additionally, Pakistan is a patriarchal society [19, 20], where most mothers are unemployed, expected to remain at home doing housechores for the extended family, excluded from decision-making, and subservient to their male partners [21]. Furthermore, all these circumstances are even more heightened in rural versus urban contexts.

### Conceptual frameworks

Two theoretical frameworks motivated this study’s conceptualization and investigation of the fathers’ roles in relation to children’s early health and development. First, the importance of fathers for promoting ECD is well-conceptualized by Bronfenbrenner’s bioecological systems theory [22]. This bioecological framework emphasizes how mothers and fathers and microsystem are one of the most proximal spheres of influence on children’s development; and moreover how broader ecological systems further interact and shape child development and parenting. Bronfenbrenner’s bioecological theory and more specifically Cabrera, Fitzgerald, Bradley, and Roggman’s [23] ecology of father-child relationships model suggests that fathers’ parenting behaviors should be examined with respect to a broad diversity of influencing factors across complex and dynamic subsystems.

Second, family systems theory hones in on the microsystem and emphasizes how multiple subsystems and relationships within the family directly and indirectly shape one another [24, 25]. Interdependence among subsystems highlights the importance of investigating the father-child relationship while also considering the contexts of father-mother relationship and also the mother-child relationship within the family. Particularly with respect to father-mother relationships, a growing body of literature has emphasized the importance of coparenting [10, 26] and how the degree of mothers’ and fathers’ functioning, coordination, and support for each other as partners additionally shape parenting and child outcomes [27–29].

### The present study

This qualitative study contributes to the global literature on parenting and ECD. We conducted 63 in-depth interviews (IDIs) and 2 focus group discussions (FGDs) with fathers and mothers of children under age-5 in a low-income, rural community of Pakistan. There were three primary research objectives of the present study: (1) to describe and compare the parenting roles of fathers and mothers; (2) to identify factors that contribute to and explain divisions in parenting roles between caregivers; and (3) to expand the concept of fatherhood by also investigating fathers’ coparenting and support for their partners. Overall, this study sought to generate new knowledge regarding how parents perceive and make meaning of paternal roles; build a conceptual model that reflects the family, social, and cultural contexts that influence paternal parenting roles and responsibilities in this specific setting of rural Pakistan; and apply these findings to inform the designs of future parenting interventions with fathers for promoting ECD.

## Methods

### Setting

This qualitative study was conducted in Naushehro Feroze district, in Sindh province, Pakistan. Naushehro Feroze is an impoverished rural community, where 32% of households are food insecure; 29% of fathers and 68% of mothers are illiterate; and children have poor health and nutrition, with 27% of children stunted in their first year of life [30]. Early learning opportunities for young children in Sindh are limited: only 7% of children under age-5 have three or more books at home and 18% of preschoolers attend an early education program [31].

### Study design and sample selection

A descriptive phenomenological, qualitative design guided this study. A phenomenological approach facilitates exploration of the essence of an experience by studying the lived experiences, perceptions, and personal meanings of individuals in the specific study context [32]. Data were collected through individual IDIs and FGDs with fathers and mothers.

Fathers were identified and recruited from January to March 2017 using an assembled list of households enumerated by the local community health worker program and the field research team. Fathers were randomly sampled from this list and contacted via phone or in-person to determine whether they met the inclusion criterion for participation: the biological father of a child under-5 years of age in Naushero Feroze district.

For IDIs, a stratified purposive sampling strategy was employed to recruit various profiles of fathers, and their partners [33]. More specifically, fathers with a child under-5 years of age and their wives were recruited based on the following characteristics: paternal education (<secondary schooling versus ≥secondary school), child gender, and child age (0–2 versus 3–5 years). A contrast sample of non-resident fathers (residing apart from family for > 1 month because of migratory labor) and their partners was also recruited to further explore variations in paternal caregiving roles. Paternal and child characteristics were collected to track the sampling progression for each stratum of the IDI sampling strategy (see Table 1). Upon visiting the home for the interview with the father, the child’s mother was also approached to determine her interest in also participating in the study. For FGDs, a separate group of fathers and their wives were recruited. More specifically, lower educated fathers and their wives were recruited because findings from the fathers IDIs suggested variability that deserved further explorations. The final study sample was determined upon ensuring that themes had reached a point of saturation, or when no new substantive information was acquired from the data [34, 35].

**Table 1:** Study sample

	Younger children (0–2 years)		Older children (3–5 years)		Any children under-5 years
	Male	Female	Male	Female	
In-depth interviews with 33 families or 63 individuals (*N* = 33 fathers; *N* = 32 mothers)					
Co-residential fathers					
Low paternal education (less than secondary school)	3	4	4	3	
High paternal education (completed secondary school or higher)	3	3	4	3	
Non-residential fathers					6
Focus group discussions with 14 individuals (*N* = 7 fathers; *N* = 7 mothers)					
One fathers group with low education					7
One mothers group with low education					7

### Data collection

The primary methodology employed was semi-structured IDIs with fathers and mothers in Sindhi using semi-structured interview guides. Questions in the IDIs were purposefully broad and included, for example: *How do you care for your young children, particularly relating to their early health and development? How do your parenting roles differ from your partner’s? How, if at all, do you (*i.e.*, child’s father) support your partner (*i.e.*, child’s mother) in caring for your young children?* Each question in the topic guide included several sub-questions and possible follow-up probes to encourage a deeper understanding of the issues at hand. While the interview guides for fathers and mothers generally covered the same content, questions focused more on understanding *fathers’* childcare roles and coparenting from both caregivers’ perspectives.

IDIs were conducted between January–March 2017 by one male and one female research assistant using the refined interview guides, with oversight and ongoing supervision from a field research manager (second author) and the first author. They received a 2-day training in research ethics, qualitative research methodologies, and interviewing techniques by the first author. For the IDIs, fathers and mothers were visited at their home and simultaneously interviewed, but separately from one another, in private settings of the home. Interviews with fathers were conducted by the male research assistant and ranged from 40 to 70 min long, while interviews with mothers were conducted at the same time by the female research assistant and ranged from 30 to 70 min.

After completing the IDIs, one FGD with fathers and one FGD with mothers were also conducted (March 2017). The FGD guide was developed specifically to better understand parental attitudes and social and cultural norms that might explain the emerging distinctions between paternal and maternal roles from the IDIs. For example, questions in the FGD included: *Why do fathers more commonly take their children out? In your opinion, do all children need both a father and mother?* For the FGDs, fathers and mothers were recruited from separate families, and not as couples. The fathers FGD was conducted by the male research assistant in a community space and lasted 90 min; and the mothers FGD was conducted by the female research assistant in one mother’s home and lasted 60 min.

IDIs and FGDs were audio-recorded, transcribed by the two research assistants who conducted the interviews, and translated into English by two independent translators unfamiliar with the research purposes of the study, immediately after the data were collected and concurrently to the completion of fieldwork (February–June 2017). The research team collectively debriefed every week through teleconference calls to discuss each week’s emerging findings or any challenges. To ultimately ensure clarity and accuracy of translations, two early ECD researchers – with prior experience working in this study context and fluency in Sindhi, Urdu, and English – reviewed a randomly selected subset (15%) of interview transcripts. Any discrepancies in translation were discussed and corrected accordingly.

### Data analysis

Data were analyzed using thematic content analysis [36]. Data were specifically analyzed by gender of the respondent. All English translated data were independently coded, annotated, and analyzed using NVivo (Version 11) software by both the first author and a graduate research assistant, who was not involved in data collection. First, an initial codebook was developed from the study objectives, research questions, and structure of interview guides. Additional codes were defined from emerging themes in the data and through consensus-building discussions between the two data analysts. Second, the two data analysts independently conducted a line-by-line, open coding of each transcript in NVivo. Third, in addition to assigning codes, the analysts documented memos while reviewing each transcript. Finally, themes were analyzed and the perspectives of fathers and mothers were triangulated to validate, compare, and contrast findings by gender of parent. Weekly meetings were held throughout the analysis process to confirm code agreement between the researchers, resolve any disagreements, review memos, and discuss emerging themes.

### Research team and reflexivity

This study was designed and supervised by the first author, a non-Pakistani male PhD candidate from the United States, trained as a global early child health and development researcher, with experience conducting qualitative research in LMICs, but no prior experience in Pakistan. The study was also supervised and managed by the second author, a Pakistani female ECD research specialist, with a master’s degree, over 10 years of relevant local and international research experience, fluency in Sindhi (the local language) and English, and from the local community herself. The second author contributed to the development of the tools and translated the tools from English to Sindhi. The interviews were conducted by one male and one female Pakistani research assistant (RA); both with master’s degrees, fluency in Sindhi and conversant in English, with previous experience with qualitative interviewing and ECD related research in Naushehro Feroze, and from the local community themselves. The male RA was a father to a young child and a husband; the female RA was single with no children. Providing research assistance to the first author, a female graduate student from the same university in the United States supported coding and analysis of the English transcripts. She is a physician from India, with 3 years of prior experience working on projects relating to ECD and early education with a local NGO in India.

During the first few weekly team debrief meetings regarding emerging findings among the first and second authors and the two Pakistani RAs, several differences in researcher perspectives became apparent, as certain findings seemed more salient to different researchers. The first author was especially interested in understanding fathers’ coparenting roles in Pakistan. The Pakistani research team initially highlighted differences between paternal and maternal roles in a seemingly obvious manner, which required additional coaching by the first author to encourage further probing and extract additional meaning from participants’ own words. During weekly team meetings, the RAs also contextualized study participants’ experiences comparing these to other relatives and community members that they personally knew, as well as their own upbringing experiences in Naushehro Feroze, Pakistan. The Indian graduate student also brought her sociocultural understanding of the South Asian context during the coding process in appreciating nuances of the rural setting that parents’ mentioned as influencing their caregiving roles. Overall, these differences in experiences, perspectives, and cultures across members of the research team and the weekly debriefs and discussions continuously throughout data collection and analysis yielded a more thorough, comprehensive, and balanced interpretation of the data by reducing individual internal biases.

### Ethical considerations

The research protocol of this qualitative study was reviewed and approved by the Institutional Review Board of the Harvard T.H. Chan School of Public Health and the Ethical Review Committee of the Aga Khan University in Pakistan. Informed consent forms were read aloud in Sindhi by trained research assistants to all participants. Participants either signed the consent forms or gave their fingerprint to indicate consent. Participants received a fruit basket and a small children’s gift (toy or book) for participating in either IDIs or FGDs.

## Results

We interviewed a total of 79 participants: 40 fathers and 39 mothers (Table 1). We conducted IDIs with 33 couples, or 33 fathers and 32 of their partners; and FGDs with 7 fathers and 7 mothers. We present the sociodemographic characteristics of the sample from the IDIs in Table 2. The FGD topic guide did not include a background characteristics section and thus detailed demographic information was not collected for each of the FGD participants. Among the IDI participants, the mean paternal age was 33.3 years, and the mean maternal age was 29.5 years. In accordance with our sampling strategy, approximately half of fathers had completed primary school (46.6%) versus secondary school or higher (53.3%). The majority of mothers (59.4%) had no education. Fathers most commonly worked in agriculture (33.3%) or some casual labor job (33.3%; e.g., miller, carpenter, driver). In particular, six fathers lived apart from their children and family for extended periods of time. Nearly half (48.5%) the children of sampled fathers were female and slightly greater than half were of the younger age-group of children aged 0–2 years (54.5%). The majority of families resided together with extended family members. We structure our results in accordance with our three primary research questions.

**Table 2:** Sociodemographic characteristics of sample from in-depth interviews with 33 families

Characteristics	Mean or N	SD or %	Range
Father characteristics (*N* = 33)			
Father’s age (years)	33.3	7.8	18–50
Father’s education			
None	4	13.3%	
Complete primary	10	33.3%	
Complete secondary	7	23.3%	
Higher than secondary	9	30.0%	
Father’s employment status			
Agriculture	11	33.3%	
Casual or day labor	11	33.3%	
Office job	4	12.1%	
Teacher	3	9.1%	
Army	2	6.1%	
Landlord	1	3.0%	
Service industry	1	3.0%	
Father residential status			
Residential	27	81.8%	
Non-residential	6	18.2%	
Mother characteristics (*N* = 32)			
Mother’s age (years)	29.5	5.9	20–40
Mother’s education			
None	19	59.4%	
Incomplete primary	2	6.3%	
Complete primary	5	15.6%	
Complete secondary	5	15.6%	
Higher than secondary	1	3.1%	
Mother’s employment status			
None	30	93.8%	
Lady health worker	1	3.1%	
Teacher	1	3.1%	
Child characteristics (N = 33)			
Child is female	16	48.5%	
Child age			
0–2 years	18	54.5%	
3–5 years	15	45.5%	
Child’s number of siblings	2	0.6	0–6
Household characteristics (*N* = 33)			
Household size	10.1	4.2	4–20
Household income (PKR)	16,717	12,192	4,000–45,000
Household composition			
Nuclear family	7	21.2%	
Joint family structure	26	78.8%	

*Note*: 1 USD is approximately 107 PKR

### Objective #1: Paternal, maternal, and shared parenting responsibilities

**Fig. 1:**
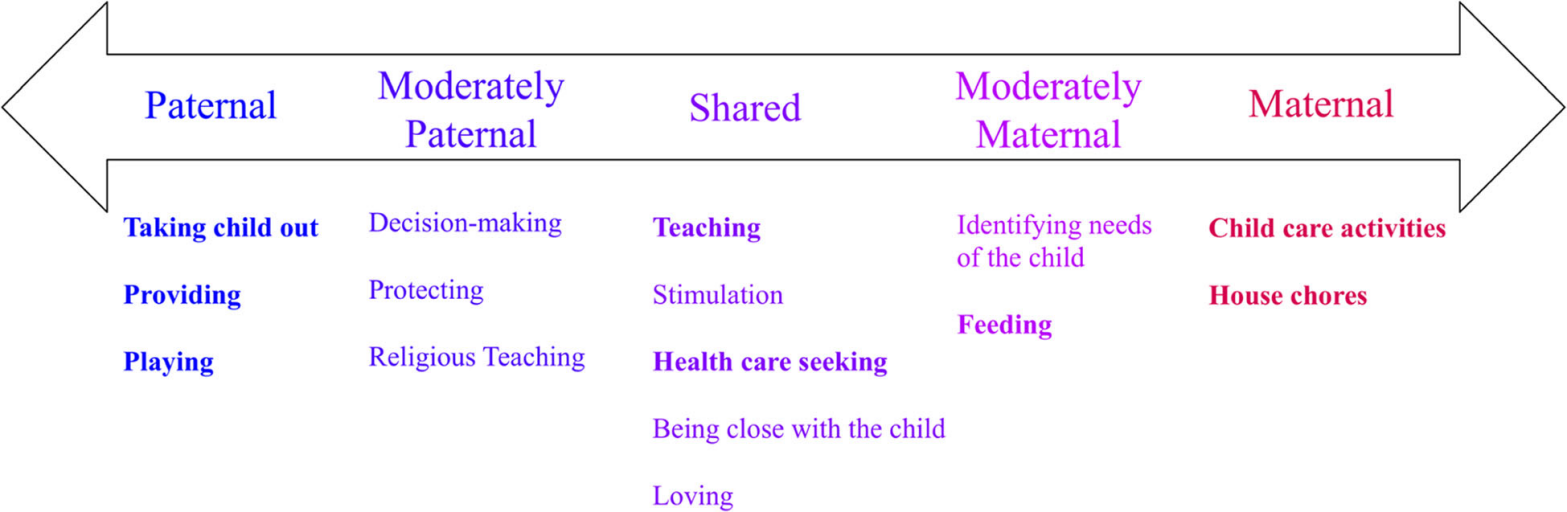
Parenting roles and responsibilities, ranging from most dominantly paternal-exclusive to most dominantly maternal-exclusive. Bolded activities represent those that were commonly reported across interviews, which are elaborated upon in the paper

The majority of fathers and mothers reported distinct gender divisions in caregiving responsibilities for young children (Fig. 1). The most dominant paternal activities were earning and providing for the child and family, taking the child on outings, and playing with the child. In contrast, parents described the most dominant maternal activities relating to basic childcare activities, house chores, and feeding. However, teaching the child, engaging in stimulation, and seeking health care were commonly mentioned as both paternal and maternal activities. We present illustrative quotes supporting each of the most salient paternal, maternal, and shared parenting roles in Table 3**.**

**Table 3:** Select illustrative quotes supporting the most salient paternal, maternal, and shared parenting roles

Themes with supporting quotes	Respondent
Paternal role	
Providing	
I go for labor and earn money, and when I come back [home] I bring important things for the child. I bring fruits, like bananas and oranges, cerelac, milk, or vegetables like ladyfinger and spinach. I get these so that he becomes healthy.	Family 25, father
Children tell him [father], “We want this, we want that”. He goes and gets things like ice cream and sometimes chips to make them happy. He takes good care of them.	Family 24, mother
Taking child out	
I take [child’s name] for outings and show her different things, and then I show her trucks and ask her what that is so that her mind works. I show her the fields and teach her the names of things, like, “This is this, you call this this.”	Family 21, father
I take him [out] so that the environment changes. When I take him out, he will see things because he gets bored at home all day, and he will have a fresh mind.	Family 32, father
When I come home he [child] says to me, “Father take me to the shop”. Then I take him to the shop. He also says that he wants to take the goats outside and play with them or give grains to the cow, so I take him there.	Family 8, father
Playing	
It’s my responsibility that I play with my child because I’m his father. I give him love and affection otherwise the child will think that my father maintains a distance from me. He’s my child.	Family 27, father
My child enjoys and also waits for me to come and play. My child gets happy and I get happy by seeing the child too.	Family 34, father
Maternal role	
Feeding	
Look, out of 24 h, a child is with his mother for 18 h. The mother feeds him, so he is closer to his mother. The mother communicates with the child… a mother’s role is more important.	Family 17, mother
...because she knows everything about the child, like what he should eat and what he shouldn’t eat. The mother knows better.	Family 6, father
Other child care activities	
Mother is responsible because she is all the time at home with the child. She knows what her child wants, what doesn’t he want, what he needs, what time will he eat, what time does he sleep, what time the child has to be cleaned, when to give him a bath. All the responsibilities are of the mother. Poor father is earning and he doesn’t know.	Family 24, mother
His mother is more responsible because she spends more time with him. From morning to 6, I am busy. During that time I come home for half an hour, but he stays with his mother more so she will be better able to observe	Family 20, father
House chores	
He does the outside work and gets everything for me. Cooking the food and feeding him is the mother’s task. A mother is at home so she will do that. She will take more care of a child. A father takes his child for an outing and fulfills his wish. I am at home so I do the chores at home.	Family 13, mother
Mother gives birth to a child, breast feeds him, brings him up, washes their clothes, makes food for them, makes him sleep on time, takes care of his neatness, meaning whatever the task is inside the home that’s a mother’s responsibility.	Family 17, father
Shared roles	
Teaching	
I sit and show him books to make him smart. I teach him from the books. I teach him words. I tell him the names of different things. I tell him that this is this color. This is a cat and this is the sound it makes. I explain to him like that. I also take him out to meet my friends so he remembers their names. My wife teaches him when I am not at home. My wife feeds him and after feeding him sits with him and plays and repeats what I have taught. She shows my books and teaches him from that. She shows him a cow and different animal and then makes him remember words and the names of animals.	Family 18, father
I talk to her as much as possible. Everyone likes a child who speaks, and my husband talks to her too. We always talk to her so that she becomes intelligent and learns to speak from childhood. He talks to her so that she becomes intelligent and her mind expands the child becomes smart with age	Family 23, mother
Health care seeking	
Mother and father have the task that first they take care of the health. The mother will tell the father what is wrong, and then they will both take him to the doctor.	Family 32, father
Whenever the child is sick, no one other than the father can take care of it. If the child gets sick he would take him to the doctor and would make his wife understand how to provide the child with medicinal dosage.	FGD, mothers
If the child gets sick then mother and father take him to the doctor and get medicine for her. Mother and father together take care of the child because God has assigned these responsibilities to mother and father.	Family 26, father

#### Paternal roles: Providing, taking the child out, and playing

All caregivers unequivocally expressed that fathers are the primary earners and providers of the households. Fathers most commonly purchased and financially provided food, clothes, play materials, school fees, and health care services for their young child. Fathers were motivated to purchase nutritious foods for their children so they could be healthier and “grow quickly”. In addition to fulfilling the needs of the child, fathers expressed wanting to fulfill their children’s desires and wishes. Parents also mentioned how children more commonly requested things from their father than mother.

Another salient paternal activity was taking the child for visits outside of the home. Parents mentioned that fathers took their child out most frequently on the weekend, or in some cases when he returned home from work in the evenings. These outings included visits to: other family members’ and neighbors’ homes, open fields, gas stations, highways, and hotels. Parents discussed how these outings provided opportunities for fathers to name places and things, for children to see and experience new things and learn from the outside environment, and meet other people.

“I want to take him to open his mind. If a car is going on its way then my son will ask, ‘Father what is that?’ So I will tell him that this is a car so he learns something. If he sees people so I tell him [who that is] so that he learns. If my son meets my friends, then he will remember their names. So in terms of memory his mind works well and he learns.” (Family 18, father)

Parents commonly explained how fathers took the child out so that her “mind expands” and for a “fresh mind”. Parents also mentioned how fathers’ taking the child out was often initiated by the child’s requesting of the father. Finally, parents highlighted taking the child out as a way of placating the child when she was crying or fussy from being at home all day.

Play was mentioned as another dominant paternal activity. Fathers commonly played hide-and-seek, marbles, cricket, and with toys with their young children. Parents discussed fathers’ play with their children as primarily a way to express love and make them happy. Moreover, some emphasized how play provided an opportunity for children to learn and engage physically, which was considered beneficial for children’s early development. In addition to the delight that children received through play, parents described how fathers enjoyed playing with their children.

#### Maternal roles: Feeding, childcare activities, and house chores

Caregivers consistently mentioned mothers as having a greater role in routine child care activities than fathers. Mothers were the primary caregiver responsible for feeding the child: breastfeeding for younger children and feeding foods to older children. Parents explained that feeding was a mother’s responsibility because fathers were out of the home throughout the day. Additionally, mothers fulfilled a variety of other care activities for the child: bathing, washing the child’s hands, dressing and changing the child’s clothes and diapers, pacifying the child, and putting the child to bed. Parents explained that daily caregiving activities were a mother’s responsibility because of her greater time spent with the child during the day, knowledge about what children needed and their preferences, and familiarity to the child’s cues.

Parents consistently emphasized how mothers were responsible for domestic house chores (e.g., cleaning, washing dishes, doing laundry). These maternal responsibilities were indirectly discussed as constraining her time and limiting her availability to simultaneously care for the child. Parents explained that house chores were mothers’ tasks because she is the caregiver who is at home all day. For instance, parents commonly delineated “in-home” responsibilities versus “out-of-home” responsibilities to describe and explain the distinctions between maternal versus paternal roles, respectively.

#### Shared paternal and maternal roles: Teaching and health-care seeking

While most parenting responsibilities were more dominantly either paternal or maternal, teaching and health care, however, were described as shared responsibilities between both caregivers. Fathers and mothers discussed their shared roles in teaching, both in terms of instilling values and engaging in cognitive stimulation with their young children. Common values that parents emphasized included respecting elders, showing kindness and not fighting with siblings or peers, and developing good manners.

The importance the Islamic faith also emerged as an important aspect of instruction, particularly beginning among preschool-aged children. Parents described encouraging their children’s early learning and recitation of the Quran and shared how fathers commonly took their preschool-aged children to the mosque. In explaining their shared roles in instilling values early on in the child’s life, parents discussed how infants model the behaviors of both parents and begin to develop their character based on how they are raised during early childhood.

Parents also highlighted how they both focused on teaching so that their children’s “little minds would open up.” For example, one father described the importance of their shared role of teaching using an analogy of nurturing a plant and building the foundation of a building:

“The first 5 years are the basis of a child’s life. He should be taught good because if someone’s roots are strong he will be good in the future… if his roots are formed in this time, he will become successful in the future… a building that is weak from the base and strong from above will not be that good that is why the first 5 years are very important.” (Family 18, father)

In particular, parents also commonly shared how they named objects and taught words, letters of the alphabet, and common greetings. Some fathers and mothers also discussed reading and telling stories to the child, though less common than naming things. Of note, several fathers and mothers qualified that the mother played a greater role in stimulation than the father because she was together with the child more throughout the day.

Caring for the child’s health also emerged as another shared responsibility of both fathers and mothers. Parents commonly described how they took the child to the doctor together, both went to get medicine for the child, or together gave medicine to the child in the event that she was sick. Interestingly, gendered distinctions were still apparent in this joint responsibility of caring for the child’s health. For instance, parents described fathers’ child health care responsibilities in terms of his financing the doctor’s visit, purchasing medicine, and physically taking the child to the clinic; whereas mothers’ roles were discussed in terms of first identifying the child’s symptoms at home, feeding the medicine, and monitoring the child’s state and recovery. In the context of seeking health care for the child, these dominant gender roles were complementary, and parents recognized and valued each other’s roles in caring for the child.

### Objective #2: Factors influencing fathers’ and mothers’ parenting roles

**Fig. 2:**
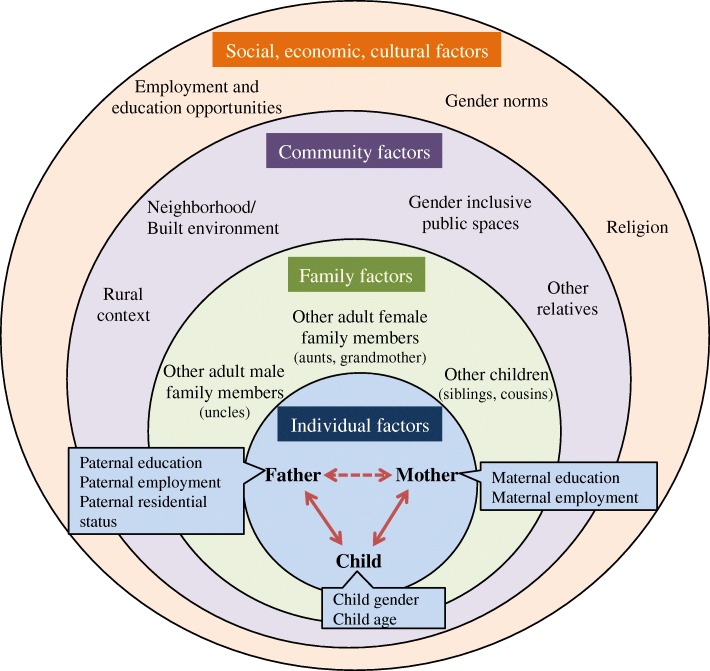
Factors that influence and explain differences in gendered parenting roles

Parents discussed a multilevel and interactive network of factors as influencing and explaining their care roles (Fig. 2). We provide several illustrative quotes supporting these factors in Table 4**.**

**Table 4:** Select illustrative quotes supporting the various factors that influence and explain paternal and maternal parenting roles

Levels and sub-themes with supporting quotes		Respondent
Individual factors		
Child age Child age	If the child is very young then in that case mother can go along with the father for doctor’s checkup. But if the child is grown up and can sit on the bike, then father can take the child with him by themselves. If the child is younger or if the child is very sick, then in that case mother can go along with his husband and child. When a child is young, he is in his mother’s lap. He is in her hands night and day. She is closest to her mother, so her mother has a greater hand.	FGD, mothers Family 26, father
Paternal residential status	I sometimes feel his absence when my husband is not here. I have to take them to the doctor and there is issue of car. Then it is difficult for me. There is money but I feel his absence that it is difficult to take to the doctors.	Family 27, mother
Paternal residential status	I am a laborer and work and live in Karachi and come after 3 months. Then whatever number of days I spend here [back in Naushehro Feroze] I try to keep my children happy and I play with them… His mother is more responsible for that [feeding the child] because I come home after two or three months… I have the role that I earn for him, fulfill his needs, the rest I do if I am at home. But right now I am not at home so his mother cares for everything... my wife is the one who plays with him and spend time together with him.	Family 15, father
Paternal employment	I am very attached to my children. My husband isn’t attached that much because he is busy. He has a job and spends most of his day busy at work. He comes back home at 7. If a man spends his whole day working and comes back in the evening, what time will he be able to give to his children? You can imagine, so taking care of them is all my responsibility. I personally feel that my children are closer to me because my husband is out most of the time.	Family 20, mother
Paternal education	Actually my wife is illiterate and I am educated. So I teach him accordingly. I want that my son becomes [educated] like the way I am. I teach him words and their pronunciation. I show him pictures in books.	Family 18, father
Maternal education	First I don’t have an education like today’s girls who do and have jobs, and I don’t have a skill. I just do house chores. I don’t know stitching or embroidery. We were poor so we don’t have an education and neither did we learn anything. If my husband is not there and doesn’t do labor or hard work, so I will need everything.	Family 24, mother
Maternal education	I say to my husband that I am illiterate you teach him how to write and read. He [the child’s father] knows everything and makes him remember and he makes children learn and writes for the child.	Family 29, mother
Extended family		
Siblings	His older brother plays with him, and they both play together 24/7. They play hide and seek or they play with toys like cars and motorcycles. And then when their father comes he also plays with them. They don’t look like father and sons, but all look like brothers.	Family 33, mother
Uncles	Yes his paternal uncle has a hand. If I am not free, then he takes him out for outing. He says, “He is crying so I am taking him out”.	Family 7, father
Uncles	He [child’s father] asks on the phone [about taking child to doctor], but most of the time I go with my brother in law because my husband is away for work most of the time.	Family 5, mother
Community		
Neighborhood	I take him to the national highway. There’s a petrol pump and he sees cars so he gets happy. Sometimes I take him to Naushero Feroze to the flour mill. He looks at the machine and how it’s working.	Family 29, father
Neighborhood	There are no children’s parks [playgrounds] near our home. There’s only one near the national highway. He takes her [child] there, but only when he has a day off from work.	Family 19, mother
Social, economic, cultural factors		
Poverty	I want to buy nice toys for him, but I am unable to because of poverty, because of not enough money. The money I get from labor is used in the house and then are all finished. So no money for children’s small necessities.	Family 28, father
Poverty	Our difficulty is that I do not have money. If I had money, children will also be happy and so will we.	Family 4, father
Gender norms Gender norms	I am a woman and a woman will only be able to see the inside environment. The husband sees the outside. But if he is not there, then the wife will not be able to raise the children on her own and purchase needs from outside. If a woman can earn at home [informally by stitching fabric], then why is there a need to go out? At home she can do stitching and earn money from home if she works hard. But other than that earning from outside is a man’s responsibility. He has more physical power than her and the hard labor that a man can do a woman cannot. There aren’t jobs here where our women can work.	Family 19, mother FGD, father

#### Individual factors

In terms of child characteristics, caregivers discussed how child age impacted both their own and their partner’s parenting roles. More specifically, parents discussed how mothers were more exclusively engaged with younger children (by breastfeeding during infancy), but how fathers became more engaged as the child grows older and is more capable of independently exploring her environment (through play). Although the majority of fathers and mothers did not express any differences in parenting roles by the gender of the child, a few caregivers mentioned certain instances of how fathers’ behaviors varied by child gender (e.g. fathers’ bathing and engaging in more physical play with boys versus girls).

Caregivers also discussed their divisions of care responsibilities with respect to several paternal characteristics: fathers’ employment, residential status, and level of education. Fathers highlighted how their employment enabled them to financially provide for the needs of their child. On the other hand, parents discussed how men’s common employment in agriculture or day laboring jobs required them to spend the majority and irregular hours of the day outside of the home, thereby limiting the direct time that men could spend together with the child.

Parents’ roles were additionally impacted by the father’s residential status. On the one hand, migratory working fathers described better paying jobs and earning more to financially provide for their child and family. On the other hand, all non-residential fathers strongly expressed how this living arrangement prevented them from caring and engaging with their child on a daily basis. For example, these fathers all mentioned how their work and residence away from the home minimized direct play interactions with their child and the opportunities for them to take their children out. Moreover, all these fathers underscored how their extended absence inextricably strained their partners’ parenting roles, by increasing the responsibilities and roles that she had to take care of to, including those that were more dominantly paternal (e.g., providing, taking the child out) to compensate for the father’s absence.

Fathers’ level of education was perceived as an important predictor of fathers’ caregiving roles. More educated fathers mentioned having the literacy and skills to teach their children how to read; they also expressed more enjoyment in stimulation activities with their child than less educated fathers. More educated fathers elaborated on how they brought home more nutritious foods for the child and explained how such balanced foods were important for the child’s health. Finally, more educated fathers also expressed a deeper understanding of ECD and articulated how early childhood was a formative period of development, during which children grew rapidly, learned good versus bad behaviors, and began building their character.

In contrast to fathers in this sample, the majority of mothers had no education. Both fathers and mothers underscored how low maternal education and maternal unemployment contributed to divisions in caregiving responsibilities and specifically mothers’ dependency on fathers for certain roles. Caregivers explicitly mentioned mothers’ illiteracy as a key explanation for why some mothers engaged less and even actively requested or relied on their partners to engage more in early learning activities, like book reading and writing. Nearly all mothers were unemployed, which also further increased their dependency on their partners’ financial provisions to meet the needs of the child and family.

#### Family factors

In our sample, the majority of children grew up with older siblings and resided with extended family members, most commonly relatives of the father’s side of the family. Parents mentioned how other family members additionally shared, supported, and even relieved fathers’ and mothers’ own caregiving roles. Of note, other family members largely conformed to the predominant gendered parenting roles delineated between fathers and mothers: with aunts and grandmothers largely supporting mothers’ roles in feeding and other child care and house chore activities and uncles primarily taking the child out and providing food or medicine for the child as needed. In particular, non-resident fathers and their partners highlight how uncles played an important role in fulfilling typical paternal caregiving roles, such as playing with the child or taking the child out, while the child’s father worked away and was apart from the family for extended periods of time.

When specifically discussing fathers’ engagement in play, a number of parents also mentioned siblings and cousins of the child, who most commonly played with the child during the day. Some parents mentioned how fathers commonly joined in when their children were playing together, while another mother mentioned how the child’s father played less with their child when he was already playing with his sibling.

#### Community factors

The rural setting impacted the type of employment undertaken by fathers in this community and relatedly the time men spent in the home. The majority of fathers worked agricultural or day laboring jobs, which involved fathers spending most of the daylight hours out of the home. Moreover, households in this rural setting were geographically far from men’s workplaces, which further contributed to fathers’ limited time at home with the child during the day. Also many fathers discussed financial instability and insecurity and related to the seasonal nature of agriculture and farming work. Many of these factors explained why non-resident fathers expressed seeking migratory work opportunities in other bigger cities for more skilled and higher-paying opportunities.

The rural context also limited the variety of places where fathers took their children out in the community. For example, in this particular community, there were few child-friendly spaces where parents could take their children to explore their environment. Only a few parents mentioned the accessibility of a playground or park; instead, parents commonly described taking their children to other family members’ homes or to visit places like open fields, a shop, the highway, a petrol station, or other commercial places.

#### Social, economic, and cultural factors

Poverty further impacted both fathers’ and mothers’ parenting capabilities as well as their emotional wellbeing. Parents underscored how the lack and instability of money was a major constraint and source of stress. Poverty undermined the role and identity of fathers as providers – with unstable jobs and sources of income causing difficulties at times for fathers to purchase food, clothes, and toys, pay for the child’s school fees, seek health care and treatment, and save money in case of emergency.

Societal gender norms also shaped parenting roles. Parents highlighted how mothers were restricted in mobility and were expected to remain at home with the child all day. Because of these social norms, mothers expressed how they were entirely reliant on fathers to fulfill the out-of-home tasks. For example, one mother shared how she was dependent on her husband to take her and their child to the doctor when one of them was ill because of how it is unacceptable for her as a woman to travel and seek health care on her own. Gender inequalities were further highlighted in terms of women’s fewer attained years of schooling and limited opportunities for employment in this community. Mothers alluded to these gender inequalities in explaining why she was illiterate and could not read to her child or financially provide for her child’s needs.

### Objective 3: How fathers coparent and support mothers as partners

In this last section, we expand our investigation of paternal parenting by exploring the dynamics between fathers and mothers and the ways in which fathers respond to and even challenge these divisions of parenting roles to support mothers and coparent. Our findings uncovered three ways by which fathers support mothers: (1) fathers can be adaptable and engage in more maternal dominant roles in times when mothers are overburdened or busy with her other responsibilities; (2) fathers discuss parenting with mothers and make decisions as a parenting team for the child; (3) fathers directly care for mothers and their own physical and emotional needs.

First, while mothers were reported as the primary caregiver who was responsible for all childcare and household tasks throughout the day, many parents also described a degree of fluidity and adaptability between fathers’ dominant caregiving roles and some other more perceived maternal roles. For instance, parents shared how fathers also feed the child, hold the child, wash and bathe the child, and even wash clothes or make the bed, particularly when mothers are preoccupied. These examples of paternal coparenting were discussed more among higher educated fathers and entirely among residential fathers. Parents highlighted several common scenarios when fathers were more likely to coparent and engage in these more maternal roles, such as when mothers were overworked with household chores or when the child started crying while she was preoccupied.

“If I am cooking food and busy when my husband comes home, then he picks them up and settles them [the children]. If they are hungry, he feeds them too. If I’m not free or I am washing the dishes or keeping the house clean, so he feeds the children. Just imagine that I’m the mother and my husband is also the mother” (Family 23, mother).

Mothers also mentioned how fathers commonly shared perceived maternal responsibilities when the mother fell ill or was too tired to complete her various childcare and home responsibilities. Again it appeared that fathers were more willing and likely to help mothers with child care-related activities than house chores.

“If the mother is sick, then it’s the father’s responsibility too that he cares of the child, feeds him food, plays with him, takes him for outings, and put on his clothes. This all helps the mother and it’s everyone’s responsibility.” (FGD, mother)

Although interview questions were specifically framed to understand how fathers supported mothers, several parents mentioned how mothers also engaged in some of the more paternal dominant roles for the child, in the event that the child’s father was away from home for long periods of time or preoccupied with his work.

“If the father isn’t free or at home, then the father’s responsibilities are fulfilled by me. If the child gets sick, I provide them with the medicine, take them to shops when the children needs clothes. All these responsibilities can be fulfilled by the mother although these are the father’s.” (FGD, mother)

Second, parents also shared how fathers actively discussed matters with their wives, problem-solved together, and provided encouragement, which in turn strengthened their individual and joint parenting roles. Couples commonly discussed purchases that the child required, parenting practices, and child health care decisions. Again, this degree of coparenting was expressed more by higher educated and residential fathers. For example, several parents shared how they as a couple discussed which foods the father should purchase for the child and family on his way home for work. For instance, one mother (Family 24) shared, “He (child’s father) asks me about their food, like what is needed and what is not. So I tell him that we need fish, vegetables, and fruits. And he gets it.” Several mothers also shared examples of how their partners encouraged them to breastfeed the child, reminded them to give doctor-recommended dosages of medicine when their child was sick, and prepare nutritious meals for the child.

Finally, parents discussed how fathers are sensitive and responsive to their partner’s physical health and emotional needs. Of note, this was the least commonly discussed aspect of coparenting and was exclusively expressed among educated and residential fathers. For example, several caregivers mentioned how fathers care for their partners’ physical health by getting medicine for her, making tea for her, and encouraging her to rest from her caregiving responsibilities. Fathers emphasized how their partner’s well-being had a direct impact on her capacity to care for their child. For instance, one father (Family 17) shared, “I make sure that my wife’s health remains good because if she is okay then she will look after the children well. But if she is not okay, how will she fulfil her responsibilities? Who will look after the children?” Moreover, some fathers also highlighted the emotional support they provided as another dimension of coparenting. For instance, one father (Family 34) shared, “…like when the child is crying, I pick him up so that her [mother] time is saved and she can relax a bit. I want that she is mentally relaxed and feels fresh too.”

## Discussion

The goal of this study was to examine fathers’ parenting roles in promoting healthy ECD in rural Pakistan. We were interested in gaining a more expansive understanding of fathers’ parenting roles both in terms of fathers’ direct relationships with their children and their indirect relationships with their partners. We found that fathers engage in both unique and shared caregiving responsibilities as mothers. Fathers also expressed multiple aspects of coparenting and ways that they worked together with their partners to care for their children. Fathers’ and mothers’ perspectives moreover illustrated how caregiving roles and coparenting relationships in rural Pakistan are further shaped by broader family, community, and sociocultural contexts.

To date, the majority of the early childhood parenting literature in LMICs has focused on the roles of mothers – from exclusive reports, experiences, and observations of mothers. Few studies have collected data from fathers themselves to understand paternal roles for their young children. The limited prior studies on fathers and ECD in LMICs have predominantly characterized men as financial providers and decision-makers for their children and families, such as in South Africa [37], Ghana [38], and Nigeria, Tanzania, and Ethiopia [39]. Moreover, previous studies have primarily suggested binary divisions in parenting roles between fathers and mothers in LMICs [40, 41].

We uncovered other salient paternal roles, such as fathers taking children out, playing, teaching, and engaging in cognitive stimulation. A strong body of literature from HICs has emphasized fathers’ play and cognitive stimulation as key dimensions of paternal engagement during early childhood [7, 42, 43]. Our findings challenge the prevailing stereotype that fathers are solely providers and decision-makers for their young children, by instead showcasing how fathers’ and mothers’ roles in contemporary rural Pakistan are more similar than mutually exclusive.

In addition to revealing a variety of other paternal roles, our study uncovered aspects of fathers’ coparenting and several ways by which fathers support their partners and work together as a parenting “team” in caring for their young child. Such coparenting was supported by fathers’ sharing of some more maternal caregiving responsibilities, communication and discussion between parents about childrearing, and fathers’ direct care and support for their partner’s own wellbeing. A growing body of literature from HICs has underscored coparenting as a critical mediating and moderating factor influencing both mothers’ and fathers’ parenting [10, 28, 44] and child outcomes [29]. Our findings are consistent with this literature and provide novel cross-cultural relevancy of Fineberg’s [10] four-domain conceptualization of coparenting (childrearing agreement, coparental support, division of labor, and joint management of family dynamics) to the rural Pakistani context.

A central aim of the study was to understand the factors that influence and explained these gendered parenting roles in our study context. We were particularly interested in exploring how paternal education and residential status shaped fathers’ roles. Our findings supported paternal education as an enabling factor of positive paternal involvement and paternal support for their partners. These findings build upon prior studies that have shown how paternal characteristics not only directly relate to fathers’ parenting, but also mothers’ parenting roles and behaviors [45].

For paternal residential status, however, findings were mixed. On the one hand, we found that non-residential fathers were less engaged in terms of time spent with their children and partners. On the other hand, we found no evidence to suggest that non-residential fathers were any less financially, emotionally, or aspirationally invested in their children. Our results are consistent with research from South Africa, which similarly found that residential status does not explain differences in fathers’ financial contributions to the child [46]. Together our findings underscore the importance of a more nuanced and inclusive conceptualization of fathers’ engagement and care – beyond residential status – to accurately reflect fathers’ parenting contribution both for their children and with respect to their partners.

Our findings also revealed a network of ecological factors interrelated within and across family, community, and sociocultural contexts that further shaped and explained fathers’ parenting roles. For example with respect to fathers taking their children out, we found that other factors such as the neighborhood environment limited the places where fathers and children visited; and broader socio-cultural norms further explained why fathers as opposed to mothers engaged in this more predominantly and freely. Our findings are largely consistent with Cabrera et al.’s [23] ecological model of fatherhood, which underscores how father-child relationships are embedded in transactional dynamic systems. Our findings extend this model developed in the United States by particularly emphasizing the significance of the extended family system in our study context of rural Pakistan. Such complex network of factors underscores the need for critical considerations of not only coparenting and the family context across diverse cultural contexts, but also community, social, economic, and cultural conditions that also shape paternal caregiving and ECD.

### Implications for interventions and future research

Findings from our study underscore the importance of parenting interventions that reflect the preferences and lived experiences of men as parents and partners. Our study suggests that programs for fathers should recognize the diversity of activities that fathers enjoy and engage in with the child to promote ECD (e.g., taking child out, playing) and equally reflect fathers’ involvement in shared caregiving roles that may be more commonly ascribed as maternal roles (e.g., stimulation, teaching). Moreover, programs should additionally strengthen coparenting dynamics and fathers’ sensitivity and support for their partners. Several recent interventions in the U.S. and U.K. have demonstrated the effectiveness of targeting coparenting on improvements in fathers’ parenting behaviors, couples relationships, and child development outcomes [47, 48].

Given the substantial amount of time fathers in rural Pakistan spend outside of the home working as the sole financial provider of the family, efforts should be made to ensure that parenting programs for fathers are designed for fathers themselves and are gender-sensitive. For example, a recent formative evaluation of a paternal parenting program in Uganda found that their structure of single-sex groups (i.e., fathers groups) facilitated by pairs of same sex facilitators (i.e, men) for the first 10 sessions, before transitioning to mixed groups that were facilitated by a pair of mixed-sex facilitators for sessions 11–20, was valued by fathers for first enabling an open and safe environment among fellow men [49]. Parenting programs with fathers will likely require a different set of ingredients in terms of content, material, and structure, from the inputs and delivery of traditional home-visiting parenting programs that have been conducted with mothers in LMICs [5].

Finally, strategies should also engage other caregivers in the extended family and intervene at the neighborhood, community, and sociocultural levels. For example, this may include community efforts to shift attitudes and norms around masculinity and gender roles in parenting, sensitization campaigns to increase awareness and understanding about the purpose of parenting programs; or more broadly, municipal investments to support parenting and ECD (e.g., child-friendly play spaces and early education centers) and national policies that redress deeply entrenched hierarchies of gender and generation (e.g., women’s employment opportunities and education for girls). For example, two recent and notable fatherhood programs in Uganda [50] and Vietnam [51] have also incorporated community campaigns, in addition to fathers group sessions, through displaying posters in the community and health centers and holding open “community celebration” events for participants to share learnings with other parents and community members and change community norms and attitudes around fathers’ parenting roles. Finally, while initiatives must target and engage men, this should not diminish women’s empowerment campaigns or efforts aimed at changing women’s gender attitudes.

### Limitations

Our study has several limitations that must be noted. First, our findings are specific to this community in rural Pakistan; consequently, results are not generalizable to other regions of Pakistan or other LMIC contexts. Second, fathers’ and mothers’ reported practices and perceptions may not necessarily correspond to actual behaviors; and moreover our findings may be subject to social desirability bias. Third, while we interviewed both fathers and mothers about coparenting, the question was framed specifically with respect to fathers’ coparenting. Consequently, results are skewed towards the ways that fathers support mothers; and less regarding how mothers support fathers.

## Conclusion

This study draws upon the lived experiences of both fathers and mothers of young children to highlight various paternal parenting roles and contextualize the environments that influence gender roles in parenting in a low-income, rural population in Pakistan. Our findings support a more expansive conceptualization and appreciation of fathers’ roles in ECD, both in terms of fathers’ direct engagements with their young children and their indirect contributions through coparenting and support for their partners. Early childhood interventions that target and include fathers as parents and partners are critically needed for harnessing gendered divisions in parenting roles and maximizing the nurturing care that children require from all caregivers to achieve their full developmental potential. Future parenting interventions should consider and additionally target the coparenting relationship as well as the various contextual factors that influence fathers’ and mothers’ parenting roles.

## Abbreviations

ECD: Early childhood development
FGDs: Focus group discussions
HICs: High-income countries
IDIs: In-depth interviews
LMICs: Low- and middle-income countries
