# Correction to: “His mind will work better with both of us”: a qualitative study on fathers’ roles and coparenting of young children in rural Pakistan

**DOI:** 10.1186/s12889-018-6271-2

**Published:** 2018-12-06

**Authors:** Joshua Jeong, Saima Siyal, Günther Fink, Dana Charles McCoy, Aisha K. Yousafzai

**Affiliations:** 1 Department of Global Health and Population, Harvard T.H. Chan School of Public Health, 665 Huntington Avenue, 11th floor, Boston, MA USA; 2 Aga Khan University, Karachi, Pakistan; 3 Swiss Tropical and Public Health Institute, Basel, Switzerland; 4 University of Basel, Basel, Switzerland; 5 Harvard Graduate School of Education, Cambridge, MA USA

**Correction to: BMC Public Health (2018) 18:1274**

**https://doi.org/10.1186/s12889-018-6143-9**

After publication of the original article [1], the authors wanted to make an amendment in the Acknowledgments section as Muneera Rasheed requested to be removed. This correction article shows the original and revised version of the “Acknowledgments”. The original article was not updated.

**Original version:** The authors thank all the individuals who participated in this study for their time and willingness to share their experiences with us. The authors thank Sadaf Lanjar and Ghullam Murtaza for data collection and transcription, Rucha Shelgikar for research assistance in data analysis, and Muneera Rasheed for assistance with translation quality checks.

**Revised version:** The authors thank all the individuals who participated in this study for their time and willingness to share their experiences with us. The authors thank Sadaf Lanjar and Ghullam Murtaza for data collection and transcription and Rucha Shelgikar for research assistance in data analysis.
